# Large-scale synthesis and self-organization of silver nanoparticles with Tween 80 as a reductant and stabilizer

**DOI:** 10.1186/1556-276X-7-612

**Published:** 2012-11-06

**Authors:** Hui-Jun Li, An-Qi Zhang, Yang Hu, Li Sui, Dong-Jin Qian, Meng Chen

**Affiliations:** 1 Shanghai Key Laboratory of Molecular Catalysis and Innovative Materials, Department of Chemistry, Fudan University, Shanghai 200433, People’s Republic of China; 2 Advanced Materials Laboratory, Fudan University, Shanghai 200433, People’s Republic of China; 3 Department of Materials Science, Fudan University, Shanghai 200433, People’s Republic of China; 4 School of Medical Instrument and Food Engineering, University of Shanghai for Science and Technology, Shanghai 200093, People’s Republic of China

**Keywords:** Silver, Nanoparticles, Tween 80, Discrete dipole approximation (DDA)

## Abstract

Tween 80 (polysorbate 80) has been used as a reducing agent and protecting agent to prepare stable water-soluble silver nanoparticles on a large scale through a one-pot process, which is simple and environmentally friendly. Silver ions can accelerate the oxidation of Tween 80 and then get reduced in the reaction process. The well-ordered arrays such as ribbon-like silver nanostructures could be obtained by adjusting the reaction conditions. High-resolution transmission electron microscopy confirms that ribbon-like silver nanostructures (approximately 50 nm in length and approximately 2 μm in width) are composed of a large number of silver nanocrystals with a size range of 2 to 3 nm. In addition, negative absorbance around 320 nm in the UV-visible spectra of silver nanoparticles has been observed, probably owing to the instability of nanosized silver colloids.

## Background

In recent years, nanoparticles (NPs) of noble metals have attracted considerable particular attention due to their unique physical and chemical properties different from those of bulk substances [1-5]. Among those noble metal nanoparticles, silver (Ag) NPs have been mostly studied owing to their high electrical and thermal conductivity as well as their extraordinary optical properties, which lead to extensive application in the fields of catalysis, electronic and optical materials [6-10]. However, the applications mentioned above depend on to a great extent the properties of Ag NPs especially on the particle shape, size, and size distribution [11-13].

There have been some shape-controlled synthetic methods of silver NPs including template-assisted methods, seed-mediated methods, polyol routes, and so on [14-20]. Different silver nanostructures such as spheres, hollow spheres, cubes, wires, rods, tubes, and plates would display different optical phenomena [21,22]. During the preparation process, silver nanoparticles can be modified and stabilized by surfactants or polymers [23-25]. Surfactants are a kind of organic compounds possessing both hydrophilic polar groups and hydrophobic nonpolar groups. Under certain conditions, the surfactant molecules can form into various structures of ordered agglomerations such as micelles, reversed micelles, microemulsions, vesicles or liquid crystals [26,27] whose microenvironment can serve as microreactors and templates for the controllable formation of nanomaterials [28-30]. On the other hand, the surface of nanocrystals is composed of different lattices, and different lattices may have different growing rates on its vertical direction. Some kinds of surfactants can stabilize certain lattice faces by hindering their growth, and the proportion of the faster ones will gradually decrease until all will disappear, making most of the surface of the nanocrystals be composed of the more slowly growing ones [31,32]. Surfactant molecules are also capable of improving the stability of system through static electricity repulsive forces, steric hindrance, and Van der Waals force by absorbing onto the surface of nanomaterials [33,34].

In most of the previous works, surfactants only acted as stabilizers or protecting agents. Actually, many nonionic surfactants, such as poly-(10)-oxyethylene oleyl ether (Brij 97, Sigma-Aldrich Co., MO, USA) [35], polyoxyethylene-(20)-sorbitan monooleate (Tween 80, Sigma-Aldrich Co.) [35], polyoxyethylene tert-octylphenyl ether (Triton X-100, Sigma-Aldrich Co.) [36], can function as reductants to synthesize noble metal nanoparticles. Premkumar et al. [37] had used Tween 80 as a reductant to react with a gold salt at room temperature and synthesized well-dispersed gold nanoparticles. Luo et al. [38] reported that silver nanoparticles were prepared under mild conditions by exploiting poly(ethylene glycol) as a reducing agent at temperature (>17°C). Debnath et al. [39] showed a solid-state high-speed vibration milling method for the synthesis of Ag NPs, in which poly(vinylpyrrolidone) (PVP) functioned as a reductant. Therefore, using those surfactants or polymers with reductive properties for the synthesis of metal nanoparticles is not only theoretically possible but also practical.

In this study, we have synthesized well-dispersed water-soluble silver nanoparticles using polysorbate 80 (Tween 80) as both the reducing agent and the protecting agent, and performed a systematic study on the formation process of silver nanoparticles, thus, produced. A trace of water in the system is very important to the homogeneity and dispersity of obtained Ag NPs. By changing the temperature, new arrays such as network- and ribbon-shaped self-organization have been observed. In addition, we consider that the formation mechanism of Tween 80-stabilized Ag NPs be similar to the oxidation of nonionic surfactants reported by Currie et al. [40]. The initial step in the oxidation of Tween 80 is the formation of a free radical in α-position to the ether oxygen. Silver ions can accelerate the oxidation process and silver nanoparticles slowly form afterwards [40].

The one-pot synthesis is clean and green, and the preparation is simple with an easy operation. Tween 80 can act as both a reducing agent and a stabilizer without adding any additional reducing agents into the reaction. Moreover, the method can provide a high concentration of nanosized silver colloids; thus, it may be developed into industrial mass production of noble metal nanoparticles.

## Methods

### Materials

Polysorbate 80 (Tween 80) and AgNO_3_ were purchased from Sinopharm Chemical Reagent Co. Ltd. (Shanghai, China) and used without further purification. Deionized water was used throughout the work.

### Synthesis of silver nanoparticles

In a typical experiment, silver particles were prepared by simply mixing AgNO_3_ in a state of solid or an aqueous solution with Tween 80. In all systems, the fixed amount of Tween 80 was 2 mL. In the aqueous solution, the silver salt solutions were prepared by dissolving an appropriate amount of AgNO_3_ in 0.2 mL of water before mixing. The solid ones are designated as the dry systems, and the aqueous solution ones the wet systems. No stirring but shaking was necessary to homogenize the solution.

The mixture was then kept at different temperatures with different reaction times. When the reaction started, the color of the solution turned from yellow to orange, and finally to dark red-brown, which indicated the formation of silver nanoparticles.

After the reaction mixture has cooled to room temperature, a large amount of absolute ethanol was poured into the mixture, which was centrifuged at 10,000 rpm for 20 min. The resulting pellet was dispersed in ethanol by gentle bath sonication, and the suspension was centrifuged again. The dispersion-centrifugation process was repeated three times to wash off the surfactants and the remaining residues. In the final step of centrifugation, the final pellet was dried and then dissolved in deionized water.

### Instruments

The optical properties of silver particles were monitored by UV-vis spectroscopy. The UV-vis absorption spectra were taken at room temperature on a UV-3150 spectrophotometer (Shimadzu, Kyoto, Japan) with a variable wavelength between 200 and 800 nm using a glass cuvette with a 1-cm optical path. A UV/visible spectrum diode-array spectrophotometer (Model HP 8453; Agilent Technologies, Palo Alto, CA) was also employed for some specific samples. All of the UV-vis absorption spectra in this paper, except those mentioned otherwise, were recorded on a UV-3150 spectrophotometer.

The selected area electron diffraction (SAED) pattern, elemental analysis, and transmission electron microscope (TEM) images were acquired on a JEOL 2100F microscope (JEOL Co., Ltd., Tokyo, Japan) operating at an accelerating voltage of 200 kV. All TEM samples were made using aqueous colloids of the metal nanoparticles directly without size selection, which were deposited onto a 230-mesh copper grid covered with Formvar.

The Fourier transform infrared (FT-IR) spectra were collected on an IRPRESTIGE-21 FT-IR spectrophotometer in the wavenumber range of 400 to 4,000 cm^−1^ at a resolution of 4 cm^−1^. The samples were prepared in the form of pellets together with KBr. The X-ray powder diffraction (XRD) pattern was recorded using a Rigaku D/max γB-ray diffractometer (Rigaku Corporation, Tokyo, Japan) in transition mode and Cu K radiation (*γ* = 1.54056 Å). Samples for measurement were prepared by dropping silver colloids on quartz plates and allowing them to dry at 40°C. The X-Ray photoelectron spectroscopy (XPS) was performed on a ESCALAB MKII X-ray photoelectron spectrometer (VG Instruments, CA, USA) using non-monochromatized Mg-Kα X-rays as the excitation source. The binding energies for the samples were calibrated by setting the measured binding energy of C 1s to 284.60 eV. Particle size distribution (PSD) analysis was carried out by manually digitizing the TEM image with Image Tool from which the average size and standard deviation of metal nanoparticles were generated.

## Results and discussions

### Characterization and study of Tween 80-stabilized silver nanoparticles

**Figure 1:**
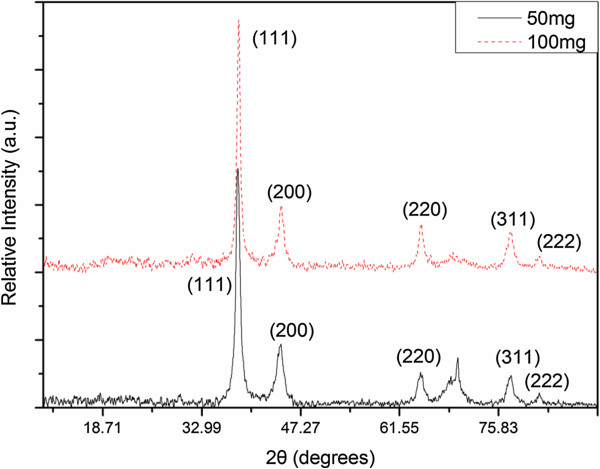
**XRD patterns of the samples prepared from the mixtures.** The mixtures include 50 and 100 mg AgNO_3_, respectively.

The XRD pattern of as-prepared nanoparticles (shown in Figure 1) confirms the formation of Ag nanoparticles with the cubic close packed (fcc) type, as in the bulk metallic Ag. Five well-resolved broad peaks, which can be indexed as (111), (200), (220), (311), and (222) diffraction peaks, are shown (JCPDS No. 4–783). The peak at 2*θ* = 69.89° belongs to the supporting silicon substrate.

**Figure 2:**
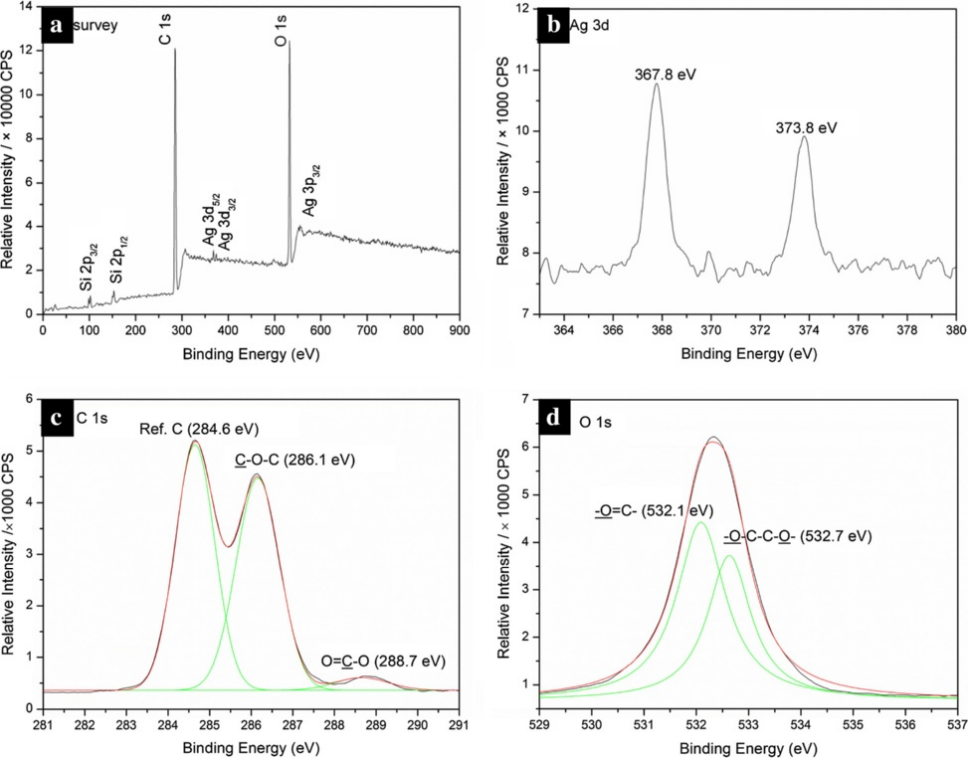
**XPS spectra of the Tween 80-stablized silver nanoparticles.** (**a**) The survey spectrum and the close-up spectra of (**b**) Ag 3d, (**c**) C 1s, and (**d**) O 1s. Red and green lines represent deconvolution of the spectra into Gaussian bands.

XPS studies have been carried out to study the chemical composition of Tween 80-stablized silver nanoparticles. Figure 2 shows the XPS spectra of obtained silver nanoparticles. The survey spectrum (shown in Figure 2a) reveals the high content of C and O, owing to the surfactant molecules attached to the surface of the nanoparticles and absorbed gaseous molecules such as oxygen and carbon dioxide [25].

Higher resolution spectra of the Ag 3d region in Figure 2b showed that the binding energies of Ag 3d5/2 and Ag 3d3/2 of the silver nanoparticles are 367.8 and 373.8 eV, respectively, with a spin-orbit separation of 6.0 eV. These results are essentially the same as those reported for the PVP-stabilized Ag nanoparticles, which indicate a strong interaction between the carboxyl oxygen atoms in the PVP chain and silver nanoparticles [41-43].

In the present study, the binding energies of Ag 3d for the Tween 80-stabilized Ag nanoparticles are between those for metal silver (368.2 eV for Ag 3d5/2 and 374.2 eV for Ag 3d3/2) and for silver (I) oxide (367.5 eV for Ag 3d5/2, 373.5 eV for Ag 3d3/2) [44]. In our opinion, this implies the strong coordination of Ag atoms with the oxygen atoms of the carbonyl (C=O) groups in the Tween 80 or oxidized Tween 80 chains. Actually Tween 80 molecules cannot provide enough carbonyl groups to interact with Ag nanoparticles, while the oxidized Tween 80 should contain a sufficient number of carbonyl groups, which further confirms the silver ions-mediated oxidative degradation of Tween 80.

The peak in the C 1s spectrum (shown in Figure 2c) can be fitted to several symmetrical peaks with binding energies of 284.6, 286.1, and 288.7 eV, which are assigned to the C 1s of the adventitious reference hydrocarbon, that of carbon –C–O–, and that of carboxyl carbon–O–C=O, respectively [45,46]. The peak of O 1s (Figure 2d) has also been deconvoluted into two bands with binding energies in agreement with those reported in the literature [41]. One of the O 1s curves located at 532.7 eV should possibly be attributed to a combination of the backbone –CH_2_***O***–CH_2_– [47], plus the oxygen in the carboxyl group (−C=***O***–) which interacted with surface of the silver nanoparticles [41].

**Figure 3:**
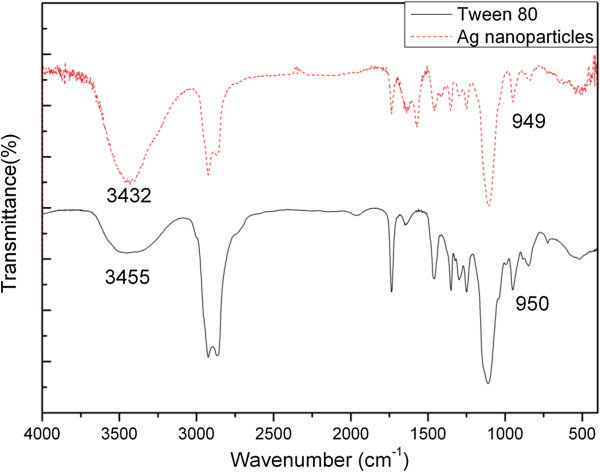
**FT-IR spectra.** Ag NPs obtained from the system added with 70 mg AgNO_3_ and pure Tween 80.

Figure 3 shows the FT-IR spectra of Ag NPs obtained from the system added with 70 mg of AgNO_3_ and the standard spectrum of neat Tween 80. A comparison of panels a and b of Figure 3 confirms that Tween 80 is absorbed on the surface of Ag nanoparticles, as both of the fingerprint absorptions are nearly identical in both spectra. As shown in the spectrum of the sample, the strong O–H and –CH_2_ stretching vibrations are represented at 3,432, 2,925 (asymmetrical stretch), and 2,863 cm^−1^ (symmetrical stretch), respectively [48-50]. The absorption at 1,458 and 1,351 cm^−1^ can be attributed to the symmetrical and asymmetrical bending vibrations of –CH_3_. The bands at 1,637 to approximately 1,654 cm^−1^ should belong to the stretching vibrations of C=C or the O–H bending mode [48-50]. The sharp and symmetric characteristic absorption peak at 1,735 cm^−1^ is due to the stretching vibration of C=O in the ester carbonyl group [48]. The absorption peak at 1,105 cm^−1^ is the stretching variation of C–O–C, and the 1,249 cm^−1^ is from the ester group.

In agreement with the XPS observation, the interaction of C=O group with the surface of Ag nanoparticles can be further proved by the FT-IR analysis. The relative intensity of the peaks (after background correction and normalization relative to the lowest value around 1,105 cm^−1^) assigned to C=O in the sample decreases significantly, possibly implying that the oxygen in the carbonyl group along the long Tween 80 chains can provide coordinative saturation of dangling bonds on the surface of the silver nanoparticles, favoring the stability of the Ag nanoparticles in water. Similar result has already been reported for Tween 80 interacting with other kinds of nanoparticles in which the decrease of the relative band intensity and even the disappearance of the C=O absorption peak around 1,735 cm^−1^ have been observed [49,50].

**Figure 4:**
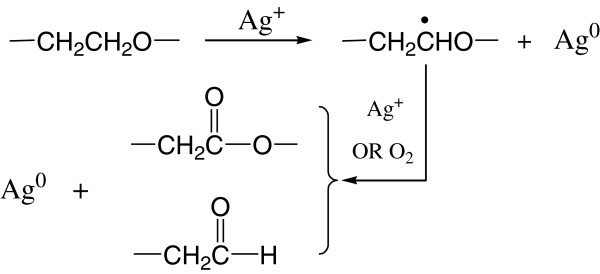
Schematic illustration of the proposed mechanism for the formation of Tween 80-stabilized silver nanoparticles.

Based on the above analysis, as well as additional relevant literature [35,37,40], we consider that the possible reduction mechanism is related to the Ag^+^-medicated oxidation of Tween 80. Tween 80, a kind of non-ionic surfactant derived from polyethoxylated sorbitan and oleic acid, is often used as emulsifier in food and pharmacological applications. Its hydrophilic groups are polyether known as polyoxyethylene (−CH_2_–CH_2_–O–) chains, which are capable of reducing metal ions to elemental metals [40,51]. As shown in Figure 4, the initial step in the oxidation of Tween 80 is the generation of a free radical in α-position to the ether oxygen, which is formed by dehydrogenation. Then, the radicals thus formed can be further oxidized into esters or degraded to form aldehydes. Noble metal ions such as Ag^+^ can accelerate the oxidation process and then get reduced.

### Concentration effects on the formation of Tween 80-stabilized silver nanoparticles

In the reaction system, there are only three kinds of reactants, AgNO_3_, Tween 80, and traces of water without additional reductants. Thus, it is convenient to evaluate the effect of the concentrations of the reactants on the products.

**Figure 5:**
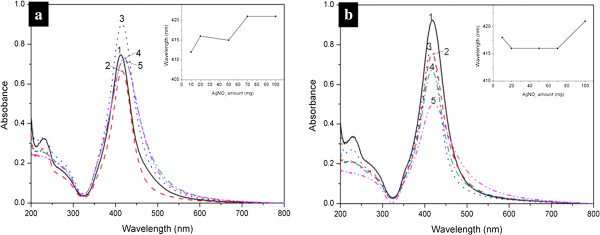
**UV-vis spectra of silver aqueous solutions obtained dry and wet systems.** The (**a**) dry and (**b**) wet systems at 100°C. 1, 2, 3, 4, 5 respectively correspond to the UV-vis spectra of systems added with 10, 20, 50, 70, and 100 mg of AgNO_3_. The inset is the diagram of wavelength of absorption peaks versus its corresponding AgNO_3_ amount.

Figure 5 shows the UV-vis spectra of the silver dispersions obtained with different concentrations of AgNO_3_ in the dry or wet system, which exhibit similar profiles with single narrow and sharp absorption peaks. The 420-nm absorption peaks correspond to the surface plasma resonance absorption of silver nanoparticles [52,53]. There are no shoulder peaks near the 350-nm absorption peaks, indicating that no bulk silver is generated [38]. Similar profiles of the absorption spectra for the silver nanoparticles, obtained in the wet system with 20, 50, and 70 mg of AgNO_3_, imply that the experimental preparation could be readily controllable and reproducible. It is worth noting that the locations of the absorption peaks, shown in the inset of Figure 5b, were essentially unchanged.

It can also be found that the increase of the amount of AgNO_3_ in the dry systems could induce a slight widening and a red shift of the characteristic peaks of silver (as shown in Figure 5a and the inset), indicating the development of larger crystalline silver nanoparticles, and that the size of particles was not uniform. This can be explained by the poor dispersing of silver nitrate in Tween 80. In the dry system, the solid silver salt could not be dispersed homogeneously in Tween 80. The unreacted salt might be gathered around the new generated silver particles and reduced on their surface, which resulted in a larger size distribution of the as-formed silver nanoparticles.

**Figure 6:**
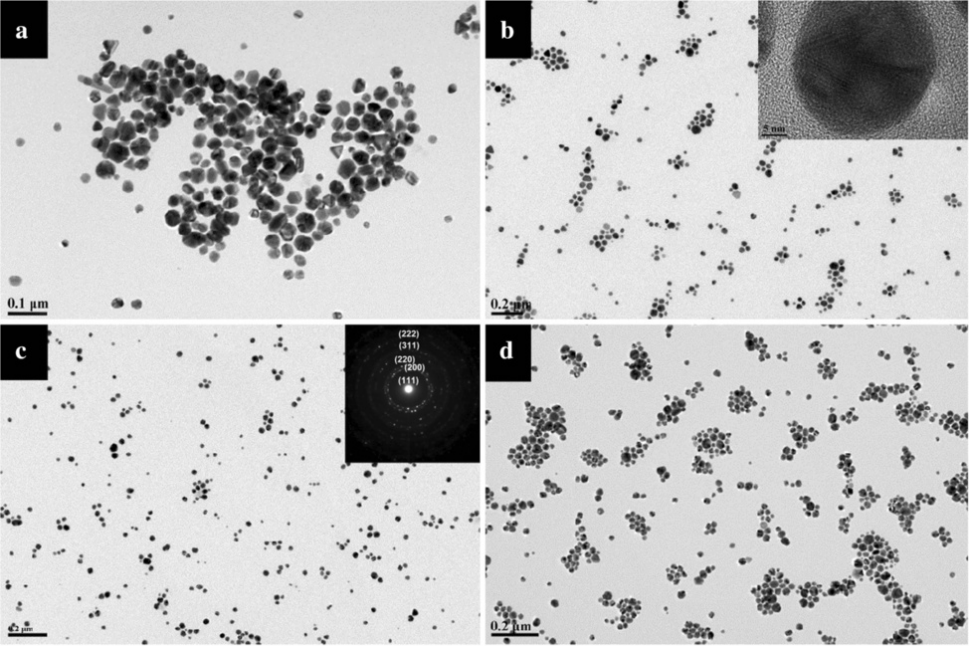
**TEM images of silver particles.** These particles were synthesized in the wet systems added with (**a**) 10, (**b**) 20, (**c**) 50, and (**d**) 100 mg of AgNO_3_ at 100°C for 3 days. The insets in (b) and (c) are the high-resolution transmission electron microscopic image and SAED patterns taken from a single particle.

Figure 6 shows the TEM images of silver nanoparticles obtained at 100°C for 3 days in the wet system. The nanoparticles in these photos appear well dispersed over a large area of the substrate. The size of silver NPs is mainly between 20 and 40 nm. Comparing the silver nanoparticles obtained in the dry system (shown in Additional file 1: Figure S1) with those in the wet system, it can be found that the wet system produced smaller nanoparticles with narrower size distribution than the dry one. The silver nanoparticles obtained in the dry system were more irregular, and some of them were joined together. While in the wet system, the silver nanoparticles were more stable with a narrower dispersity. These differences can easily be explained by the reason that silver salt in the wet system can react with Tween 80 more effectively than the dry system. In addition, the AgNO_3_ concentrations show no significant impact on the morphology of the formed Ag particles.

**Figure 7:**
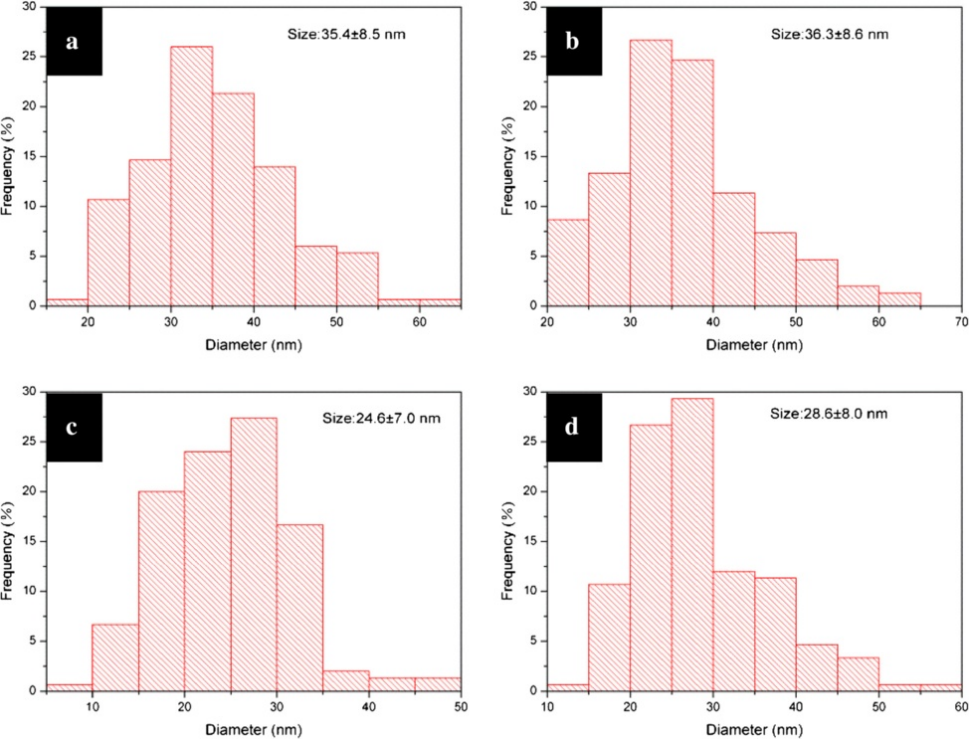
**PSDs of silver particles.** Ag particles were synthesized in the wet systems added with (**a**) 10, (**b**) 20, (**c**) 50, and (**d**) 100 mg of AgNO_3_ at 100°C for 3 days.

Figure 7 is the corresponding PSD of the nanoparticles shown in Figure 6. It is obvious that the increase of the amount of AgNO_3_ could induce the decrease of particle size. A larger amount of silver salts tends to produce more nuclei in the first stage, and the growth rate of the silver particles is more rapid [54]. The system with a smaller amount of AgNO_3_ might endure Ostwald ripening, leading to an increase of particle size. Moreover, when mixing 100 mg AgNO_3_ with 2 mL Tween 80, that is to say, the synthetic concentration of silver ions was up to 0.29 M, the obtained system could still remain stable and yield comparatively well-dispersed nanoparticles.

The insets are the high-resolution transmission electron microscopy image (HRTEM, shown in Figure 6b) of a nanoparticle and SAED patterns (shown in Figure 6c) taken from silver nanoparticles. The HRTEM gives a well-resolved fringe spacing (0.23 nm), which agrees well with the spacing of the (111) lattice places of silver nanoparticles, and indicates that the nanoparticles are single nanocrystals. From the SAED image, we can see some diffraction rings respectively corresponding to the different lattice of a centered cubic silver nanoparticle that is (111), (200), (220), (311), and (222).

### Self-organization of Tween 80-stabilized silver nanoparticles

A correlation between the dissolution rate of the surfactant and the temperature has already been proved to exist [55]. In order to study the effects of temperature on the system, we have carried out the reactions at 90°C and 110°C without changing any other conditions.

**Figure 8:**
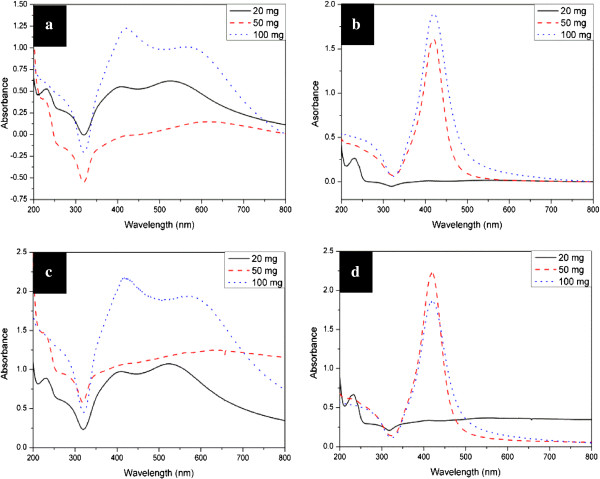
**UV-vis spectra of aqueous solutions of silver nanoparticles synthesized at different temperatures.** (**a**) and (**c**) 90°C, (**b**) and (**d**) 110°C. The spectra of (**a**) and (**b**) were acquired on a UV-3150 spectrophotometer, while (**c**) and (**d**) on a diode-array spectrophotometer.

Figure 8 shows the UV-vis extinction spectra of aqueous suspensions of silver nanoparticles produced with different concentrations at different reaction temperatures. The absorption peaks of the suspension obtained at 90°C in the wet system are wide, and the absorbance around 320 nm is below zero (shown in Figure 8a). We have repeated the process including the synthesis and characterization under the same conditions and always obtained the negative data for the suspension. Careful inspection reveals that the negative absorbance can probably be attributed to the instability of the nanoparticles, which was proved by the perceptible slow precipitation during the testing process. To further address the issue of the negative absorbance in this system, the UV-vis spectra (shown in Figure 8c,d) of the same samples were also recorded on a diode-array spectrophotometer (model HP 8453, Agilent Technologies, CA, USA) with scan rate of 100 ms. The profiles of the as-obtained UV-vis spectra (Figure 8c,d) of the silver colloids are almost identical to those (Figure 8a,b) acquired on a UV-3150 spectrophotometer. However, all the intensities of the absorption obtained with fast scan rate of 100 ms are positive, which further implies that negative absorbance around 320 nm acquired on a UV-3150 spectrophotometer indicates the instability of the as-produced Ag nanoparticles. The correlation may be used as an indicator of the instability of silver colloids by varying the scan rate, although there has been no report about negative absorbance in the UV-vis absorption spectrum of the Ag nanoparticles. Li and Xia have reported a negative absorption of light from the cubic gold nanosystem with a gain material [56], which is different from our study.

The Ag nanoparticles, produced in the wet system with 50 and 100 mg of AgNO_3_ at 90°C, show two absorption peaks around 410 and 560 nm, respectively, with a long tail at the long-wavelength side of the band (shown in Figure 8a,c). The absorption features are in good agreement with the results for the aggregation of polymer-coated silver nanoparticles [24] or the salt-induced silver aggregates [57,58]. In addition, the red shift of the band in the long-wavelength side indicates that the size of the silver aggregates increase with the concentration of AgNO_3_[57,58].

On the other hand, the absorption spectra of the Ag nanoparticles, obtained with 50 and 100 mg of AgNO_3_ at 110°C in the wet system, exhibit a single sharp, symmetrical peak around 420 nm. While the Ag nanoparticles produced with 20 mg of AgNO_3_ show an absorption profile with very low absorbance and similar to those observed for silver nanoparticles produced at 90°C, presumably owing to the aggregation of Ag nanoparticles. The silver nanoparticles generated with 20 mg of AgNO_3_ were more instable than those obtained with high concentration of silver salt, which has also been observed in the silver nanoparticle production at relative lower temperature. The observed behavior can be explained by the slow initial nucleation rate. In detailed speaking, the fewer silver salts existed in the starting stage, the slower the nucleation process occurred, and the fewer nuclei were generated in the nucleation stage. The slow nucleation rate and the fewer nuclei result in the larger-size distribution of Ag nanoparticles, followed by the instability of silver colloids.

**Figure 9:**
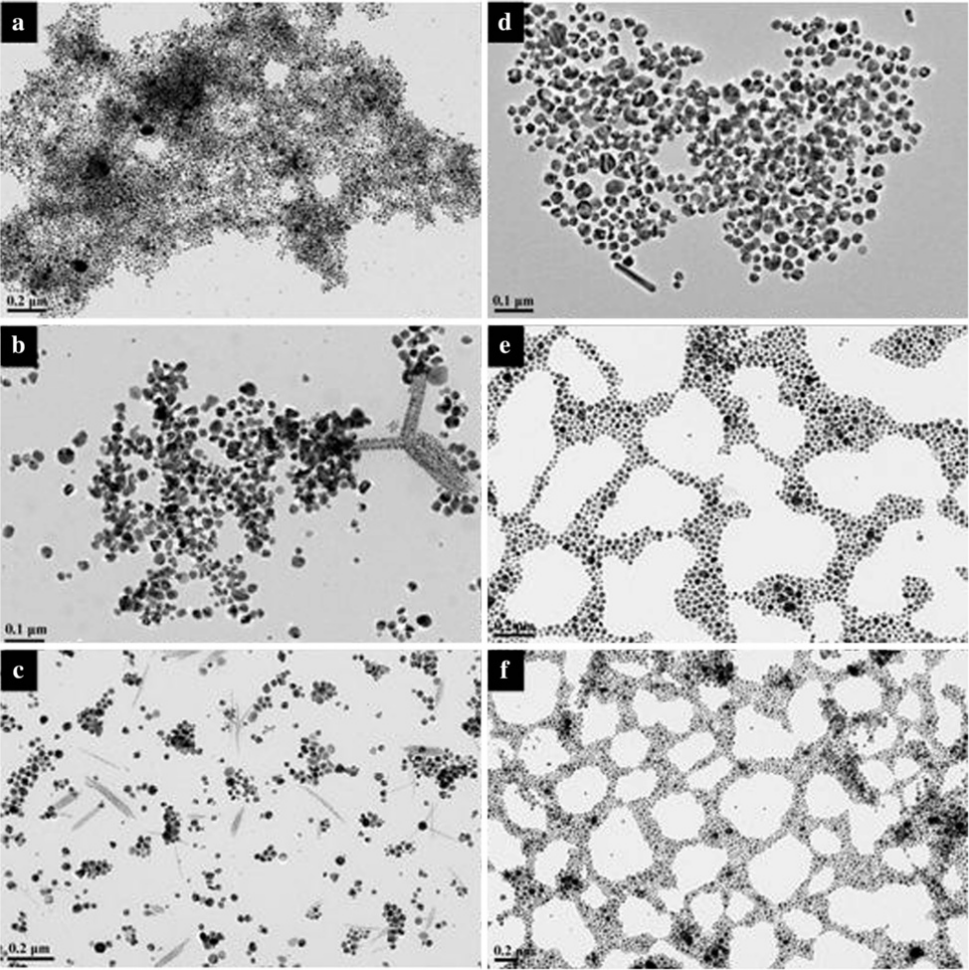
**TEM images of silver nanoparticles obtained in wet systems.** (**a**) 20, (**b**) 50, and (**c**) 100 mg of AgNO_3_ at 90°C; (**d**) 20, (**e**) 50, and (**f**) 100 mg of AgNO_3_ at 110°C for 3 days.

To better understand the effect of temperature on the morphology of silver particles, the TEM characterization has been done. Figure 9 shows the TEM images of the silver particles in wet systems at different temperatures. Some interesting arrays of particles could be clearly seen from the images. When the temperature is 90°C, a few one-dimensional spindle-shaped aggregates consisting of roughly close-packed silver nanoparticles of about 10 nm could be found in Figure 9b,c. These aggregates are relatively rigid, around 15 to 300 nm in length and 20 to 30 nm in width. Similar aggregates have been reported for the surfactant-stabilized nanoparticles, such as BaSO_4_[59] and hematite [60]. In the present study, the surfactant concentration is very high; some of the surfactant aggregates or micelles may be deformed and can stay as different shapes at relatively low temperature, thus making it possible to generate different nanoparticle aggregates [61].

Then, in Figure 9d, a mixture of different shapes of particles is observed, making sense the particular profile of its absorption peak. Moreover, when the silver salts with high concentration were used in the system at 110°C, the obtained silver nanoparticles have a tendency to self-organize into 2D well-ordered arrays. The formation of these nanoparticle arrays may be attributed to the synergetic effects of interparticle, particle-substrate, and solvent-substrate (wetting) interactions. When the solvent began to evaporate during the sample preparation for TEM characterization, the solvent film forming on the surface of the copper grid became unstable, and small droplets generated. The particles contained in the droplets would be left behind fast at the margin of the merge point after ethanol and would be entirely evaporated, which finally caused the formation of nanonetworks [62]. On the other hand, strong adsorption of dendritic-structured Tween 80 on silver nanoparticles facilitates the cohesive interaction between the particles such as interdigitation and interpenetration [63], favoring the self-assembly of Ag nanoparticles. Similar arrays, including some hexagonal close-packed arrays, of noble metal nanoparticles have been reported before [64,65].

**Figure 10:**
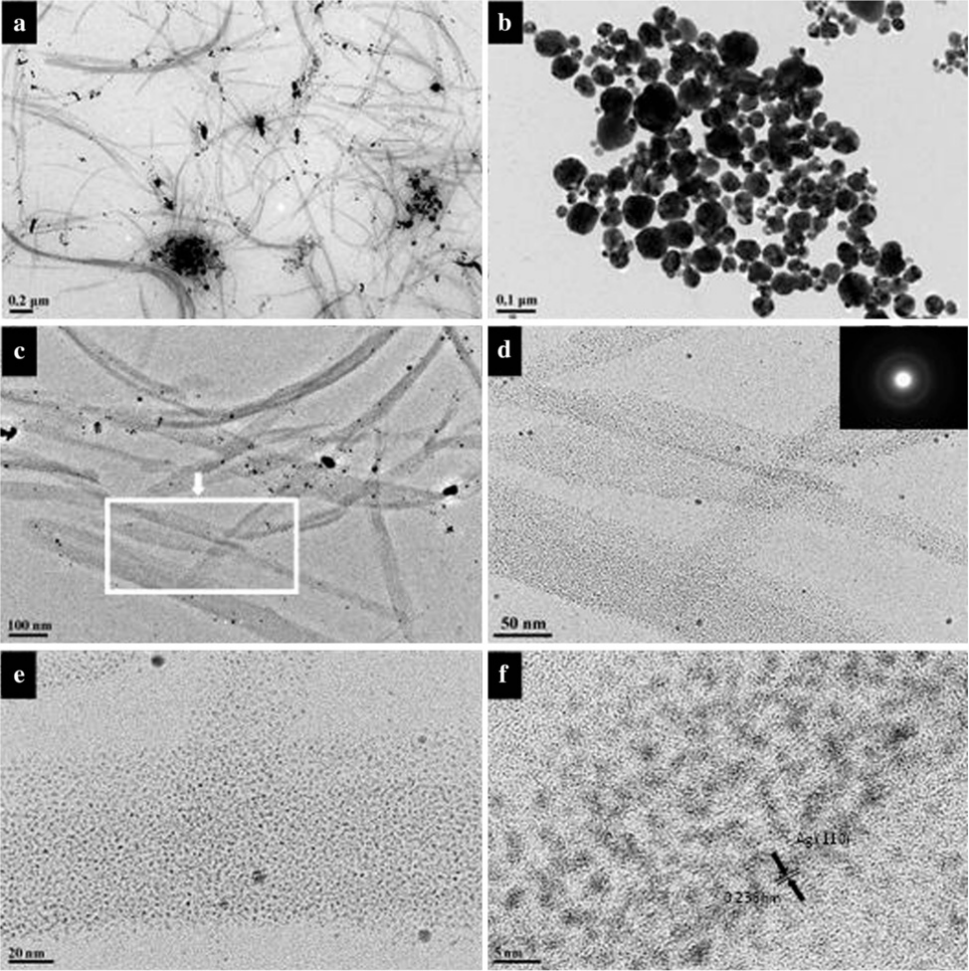
**TEM images of silver nanoparticles.** Obtained in (**a**) wet systems; (**b**) dry system with 50 mg of AgNO_3_ at 90°C for 1 day. (**c**) to (**f**) are the images with higher magnification. The insets are the ED patterns of the ribbons.

To investigate the effect of reaction time on the aggregate shape of silver nanoparticles and to better understand the evolution process of nanoparticle aggregates, we varied the reaction time from 1 to 4 days at 90°C. The experimental results reveal that shorter time could produce silver ribbons (shown in Figure 10). It is noteworthy that many ribbons are twisted or folded, implying a good flexibility. There are also a few large particles attached on the ribbons. Careful investigation with HRTEM reveals that the ribbon mainly consists of a large number of tiny silver nanoparticles with a size range of 3 to 4 nm (Figure 10f). The inset in Figure 10d also confirms that the nanoparticles are crystals. When the reaction time is longer, the silver nanoparticles would like to assemble into rigid spindles (shown in Additional file 2: Figure S2). Researchers found that the presence of a lamellar liquid crystalline phase might play an important role in the formation of the silver nanoparticle ribbons because the nanometer-sized water layers confined the packing of the particles in the direction perpendicular to the water layers, leading to the ribbon packing of the nanoparticles [66]. This conclusion is proved to be reasonable by the TEM images of silver nanoparticles synthesized in a dry system (shown in Figure 10b).

A plausible formation process for the silver ribbons is briefly presented as following: It is more likely that the concentrated Tween 80 molecules aggregated into one-dimensional micelles in the presence of silver ions. When the temperature reached about 90°C, a large number of tiny silver nanoparticles generated quickly around the micelles of Tween 80. Subsequently, the tiny silver nanocrystals started to form aggregates, most of them ordering in ribbon shape. The silver nanocrystals could also act as the cross-linker of Tween 80 molecules, which kept the ribbon-shaped aggregates in water after purification. As the reaction proceeded, some tiny particles grew larger because of coalescing or Ostwald ripening. More and more discrete silver nanoparticles with the size of 20 nm or so appeared in the products obtained in the three- or four-day reaction. Only a small fraction of nanoparticles, which had not grown into larger ones, tended to form into quite rigid spindles with bits of stabilizers around them (see Additional file 2: Figure S2).

### Theoretical studies of the dependence of the UV-vis spectra upon aggregate shape and size of Tween 80-stabilized silver nanoparticles

Based on TEM data about the size of silver nanoparticles and their aggregate shape, we use computer simulations to see whether the real UV-vis spectra corresponded well with the theoretical calculations. The UV-vis spectra show the extinction efficiency of the obtained product. The relation of extinction, scattering, and absorption efficiency factors are as follows:

$$Q_{\text{ext}}=Q_{\text{sca}}+Q_{\text{abs}}\text{.}$$

The simulation methods of calculating the absorption and scattering efficiency usually belong to two categories: the exact and approximated solutions [4]. In this paper, we use DDSCAT7.2 [67], an open-source Fortran 90 software applying the discrete dipole approximation (DDA) [68], to calculate scattering and absorption of electromagnetic waves by targets with arbitrary geometries and complex refractive index.

In our experiment, we have synthesized Ag nanoparticles coated with Tween 80. According to the TEM images of the products, some silver nanoparticles aggregated to form one-dimensional spindle-like structures. The structure was a mixture of large amount of silver nanoparticles and organic coat, so that we may consider it as silver-Tween 80 alloy. Besides, the solution we obtained also contained large amount of dispersive Ag nanoparticles and aggregated molecules of Tween 80. Thus, we established a model this way: calculate the extinction efficiency of a Ag-Tween 80 alloy ellipsoid (Ag/Tween 80 = 7:3), whose ratio of the major axis to minor axis equals to 2:1. Then, mix it with Ag-Tween 80 alloy sphere particles (Ag/Tween 80 = 8:2). Initial parameters are set as effective radius of ellipsoid = 25 nm, effective radius of sphere = 10 nm, and refractive index of ambient medium = 1.33 (H_2_O).

For homogeneous Ag-Tween 80 alloy particles, the dielectric constants can be calculated as follows [69-71]:

$$\in_{\text{Alloy}}\left(x_{\text{Ag}},\text{ω}\right)=x_{\text{Ag}}\in_{\text{Ag}}\left(\text{ω}\right)+\left(1-x_{\text{Ag}}\right)\in_{\text{Tween}-80}\left(\text{ω}\right)\text{,}$$

where *x*_Ag_ means the Ag fraction of Ag-Tween 80 alloy; ω means the frequency of incident light.

The relationship between dielectric constant and refractive index can be described as follows:

$$\in \left(\text{ω}\right)=n{\left(\text{ω}\right)}^{2}\text{,}$$

where *n* means complex refractive index.

**Figure 11:**
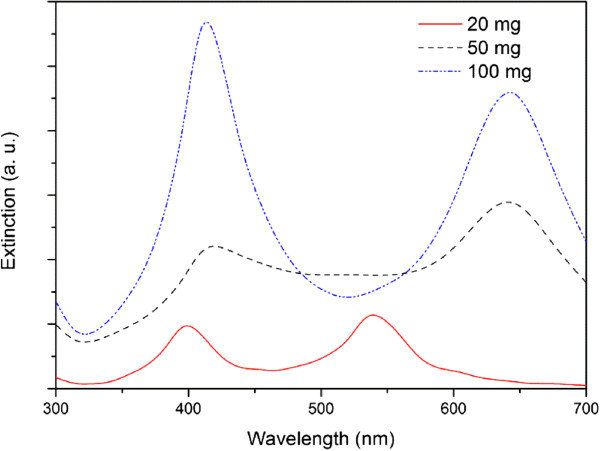
**Simulated absorption curves of silver nanoparticles obtained with different AgNO**_**3**_**concentrations.**

By simply calculating the dielectric constants of Ag-Tween 80 alloy, we can get its refractive index table as the input file. Figure 11 shows the simulation results for the silver nanoparticles obtained from the starting solution with AgNO_3_ concentration of 20, 50, and 100 mg. The extinction curve shows two peaks, appearing around 400 and 500 to 700 nm, respectively. This simulation result corresponds well with the real UV-vis spectra of the silver nanoparticles with one-dimensional aggregates (shown in Figure 8a,c).

Figure 11 also shows the simulation results of extinction efficiency of the mixed system when the fraction of ellipsoid was decreased while the fraction of sphere was increased. By increasing the fraction of the sphere particles, the extinction intensity of the peak at ca. 410 nm grows a lot. It illustrates the shape of the UV-vis spectra when the amount of A_g_NO_3_ equals to 100 mg.

The extinction spectra in Figure 11 show more distinct profiles, compared with the real absorption spectra of the silver nanoparticles (shown in Figure 8a,c). We found in the simulation that if more Ag-Tween 80 alloy sphere particles in the simulation model, the extinction peaks tend to be less distinct. That is because alloy particles have a larger complex refractive index than the ambient environment, water, but smaller than the complex refractive index of pure silver; thus, the extinction peak appears at the middle area. In addition, we use an ideal model of nanoparticles with uniform size; while the sizes of real silver nanoparticles vary at some extent, this also leads to gradually expanded peaks. Moreover, in the real mixture system, large amounts of Ag nanoparticles, together with Tween 80, are dispersed randomly, making it more difficult to separate these extinction peaks. Similar profile differences between the absorption spectra of the real nanoparticles and the simulated data have also been reported [72,73].

## Conclusions

In summary, we have prepared well-dispersed stabilized silver nanoparticles in which Tween 80 acts as both stabilizer and reductant. Special arrays and aggregates of nanoparticles were found and thoroughly studied, and a possible mechanism for the formation of silver nanoparticles has been proposed. The optical absorption obtained by DDA simulation for the aggregated silver nanoparticles was in good agreement with those from experiments. The one-pot synthesis is clean and easily operated which may be applied to some industrial demands.

## Competing interests

The authors declare that they have no competing interests.

## Authors’ contributions

HJL carried out the synthesis and characterizations of the products, and drafted the manuscript. AQZ carried out the theoretical simulations of UV-vis spectra. YH carried out the experiments. LS, DJQ, and MC contributed in the design and discussion of this work, and in the revision of the manuscript. All authors read and approved the final manuscript.

## Supplementary Material

Additional file 1 **Figure S1. **TEM images of silver particles in dry systems adding with (a) 10, (b) 20, (c) 50 and (d) 100 mg AgNO_3._ The insets in (b) and (c) are HRTEM image and electron diffraction patterns taken from a single particle. Click here for file

Additional file 2 **Figure S2. **TEM images of silver particles in wet systems adding with 50 mg AgNO_3_ at 90°C for (a) 3 and (c) 4 days. (b) and (d) are the magnified images of the nanoparticle arrays shown in (a) and (c), respectively. Click here for file
