# Supplementary material for: Large-scale synthesis and self-organization of silver nanoparticles with Tween 80 as a reductant and stabilizer
Source: Nanoscale Res Lett. 2012 Nov 6;7(1):612. doi: 10.1186/1556-276X-7-612 (PMC3503618; doi:10.1186/1556-276X-7-612)
Supplement: Additional file 1 — Figure S1. TEM images of silver particles in dry systems adding with (a) 10, (b) 20, (c) 50 and (d) 100 mg AgNO3. The insets in (b) and (c) are HRTEM image and electron diffraction patterns taken from a single particle. [file 1556-276X-7-612-S1.doc]

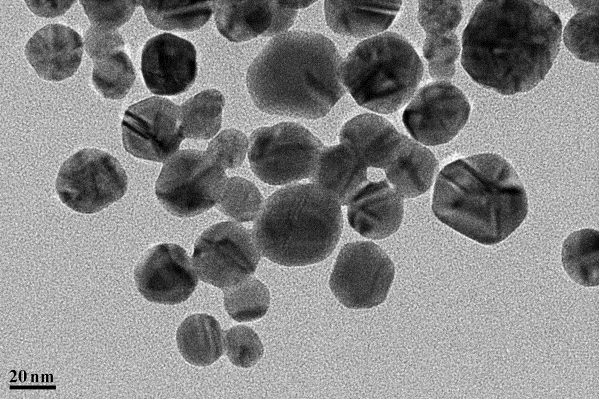

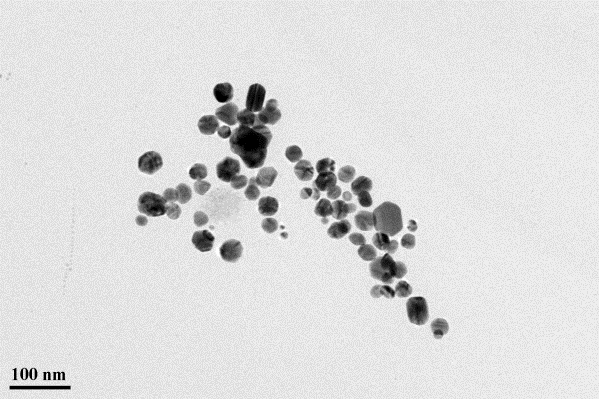

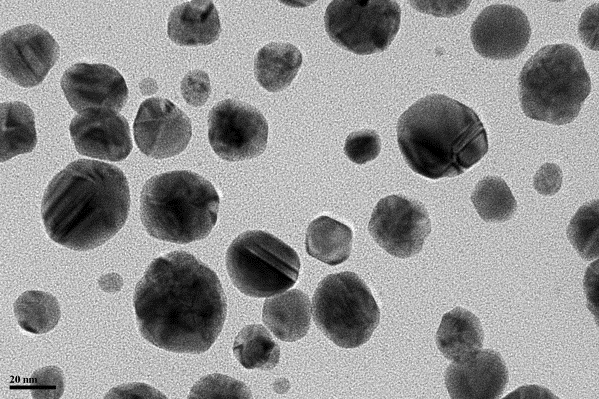

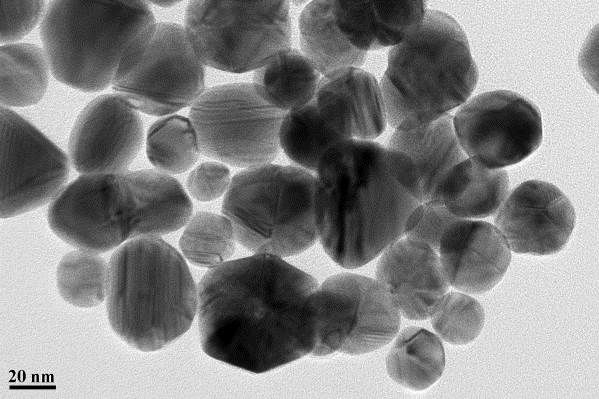


**d**

**c**

**b**

**a**

**Figure S1.** TEM images of silver particles in dry systems adding with (a) 10 mg, (b) 20 mg, (c) 50 mg and (d) 100 mg AgNO_3_. The insets in b and c are HRTEM image and electron diffraction patterns taken from a single particle.
