# Supplementary material for: Large-scale synthesis and self-organization of silver nanoparticles with Tween 80 as a reductant and stabilizer
Source: Nanoscale Res Lett. 2012 Nov 6;7(1):612. doi: 10.1186/1556-276X-7-612 (PMC3503618; doi:10.1186/1556-276X-7-612)
Supplement: Additional file 2 — Figure S2. TEM images of silver particles in wet systems adding with 50 mg AgNO3 at 90°C for (a) 3 and (c) 4 days. (b) and (d) are the magnified images of the nanoparticle arrays shown in (a) and (c), respectively. [file 1556-276X-7-612-S2.doc]

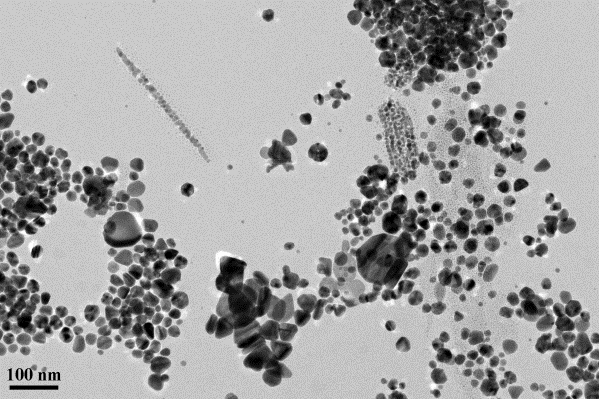

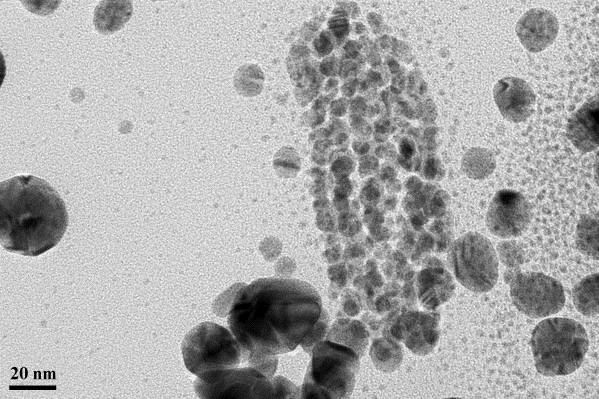

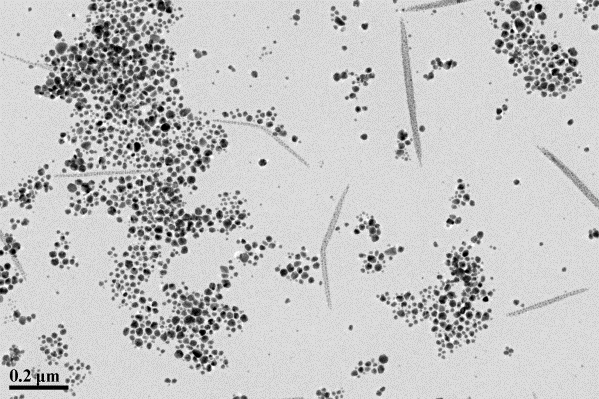

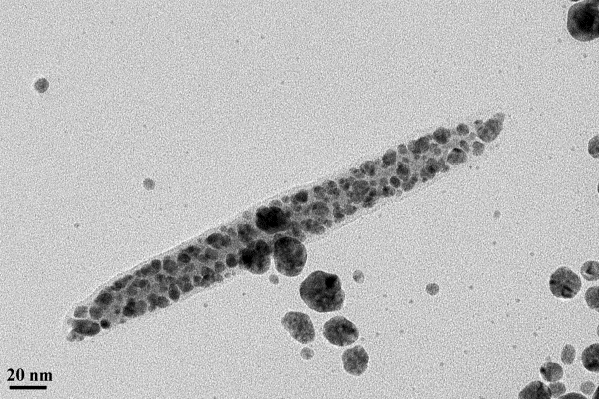


**a**

**d**

**b**

**c**

**Figure S2.** TEM images of silver particles in wet systems adding with 50 mg AgNO_3_ at 90 °C for (a) 3 days and (c) 4 days. (b) and (d) are the magnified image of the specular arrays in the left.
